# Repurposing Dipyridamole in Niemann Pick Type C Disease: A Proof of Concept Study

**DOI:** 10.3390/ijms23073456

**Published:** 2022-03-22

**Authors:** Rita Pepponi, Roberta De Simone, Chiara De Nuccio, Sergio Visentin, Andrea Matteucci, Antonietta Bernardo, Patrizia Popoli, Antonella Ferrante

**Affiliations:** 1 National Center for Drug Research and Evaluation, Istituto Superiore di Sanità, Viale Regina Elena 299, 00161 Rome, Italy; rita.pepponi@iss.it (R.P.); roberta.desimone@iss.it (R.D.S.); sergio.visentin@iss.it (S.V.); andrea.matteucci@iss.it (A.M.); antonietta.bernardo@iss.it (A.B.); patrizia.popoli@iss.it (P.P.); 2 Research Coordination and Support Service, Istituto Superiore di Sanità, Viale Regina Elena 299, 00161 Rome, Italy; chiara.denuccio@iss.it

**Keywords:** Niemann Pick type C disease, adenosine, A_2A_ receptor, dipyridamole

## Abstract

Niemann Pick type C disease (NPC) is a rare disorder characterized by lysosomal lipid accumulation that damages peripheral organs and the central nervous system. Currently, only miglustat is authorized for NPC treatment in Europe, and thus the identification of new therapies is necessary. The hypothesis addressed in this study is that increasing adenosine levels may represent a new therapeutic approach for NPC. In fact, a reduced level of adenosine has been shown in the brain of animal models of NPC; moreover, the compound T1-11, which is able to weakly stimulate A_2A_ receptor and to increase adenosine levels by blocking the equilibrative nucleoside transporter ENT1, significantly ameliorated the pathological phenotype and extended the survival in a mouse model of the disease. To test our hypothesis, fibroblasts from NPC1 patients were treated with dipyridamole, a clinically-approved drug with inhibitory activity towards ENT1. Dipyridamole significantly reduced cholesterol accumulation in fibroblasts and rescued mitochondrial deficits; the mechanism elicited by dipyridamole relies on activation of the adenosine A_2A_R subtype subsequent to the increased levels of extracellular adenosine due to the inhibition of ENT1. In conclusion, our results provide the proof of concept that targeting adenosine tone could be beneficial in NPC.

## 1. Introduction

Niemann Pick type C (NPC) disease is a genetic disorder with an estimated incidence of ~1:100,000 [1,2]. It can be caused by mutations in either the *NPC1* (95% of the cases) or *NPC2* genetic locus; the NPC1 protein is located in the membrane of late endo/lysosomes (LE/L) while NPC2 is a soluble endo/lysosomal protein acting in sequence with NPC1 to bind and transport cholesterol out of the LE/L compartment [3]. Mutations in one of these genes impair intracellular cholesterol trafficking and homeostasis, leading to the accumulation of unesterified cholesterol, sphingolipids, and other lipids in the endosomal/lysosomal system of cells of various tissues, including the brain. These defects in the storage and transport of lipids trigger a cascade of pathological events that are responsible for the multisite pathology characteristic of this disorder [4]. Although peripheral organs are affected, with spleen and liver very often found abnormally enlarged, disease severity is defined by the neurological involvement due to the central nervous system (CNS) pathology. Indeed, the neurodegeneration due to the massive loss of Purkinje neurons in the cerebellum and the diffuse atrophy in other brain regions such as the hippocampus [5], together with neuroinflammation and dysmyelination, are the main causes of the severe neurological symptoms typical of NPC (ataxia, tremor, epilepsy, dystonia, progressive dementia, and depression) [6].

So far, the only drug approved in the EU for the treatment of progressive neurological manifestations in NPC patients is miglustat, a disease-modifying agent that can improve clinical signs of the disease [7]; while a key effect of miglustat on glycosphingolipid metabolism has been suggested, its precise mechanism of action is not yet fully understood [7]. Recently, the heat-shock protein-amplifier arimoclomol obtained the Breakthrough Therapy Designation from the American Food and Drug Administration [8]. Given the very limited therapeutic options for NPC, and the lack of curative treatments, an unmet medical need exists.

Adenosine is a nucleoside distributed throughout the body and a paracrine homeostatic modulator of different cellular functions [9]. Adenosine levels are finely tuned by the orchestrated action of enzymes including adenosine deaminase (ADA), which degrades adenosine in inosine, and adenosine kinase (ADK), which converts adenosine into adenosine-monophosphate (AMP). Once formed, adenosine can be transported across cellular plasma membranes by bidirectional equilibrative nucleoside transporters (ENTs) according to its concentration gradient across the membrane; alternatively, extracellular adenosine can be generated by the hydrolysis of locally released adenosine triphosphate (ATP) from neurons or astrocytes through the activation of ectonucleotidases present in the plasma membrane such as CD73 [10,11,12,13]. Physiological levels of extracellular adenosine are necessary to properly transduce signaling pathways mediated by the G-protein-coupled adenosine receptors A_1_, A_2A_, A_2B_, A_3_ [14]. In the CNS, adenosine plays an important role in controlling synaptic plasticity, cognition, sleep, motor function, and neuronal survival [14]. This implies that dysfunction in its levels might lead to some neurological impairments; concurrently, reduced adenosine levels have been observed in disorders such as Alzheimer’s and Huntington’s diseases and enhancing adenosine tone through ENT1 blockade rescued some pathological hallmarks in in vivo models of these diseases [15,16]. 

In NPC, a reduced extracellular adenosine level has also been shown in the brain of a mouse model of the disease (NPC1^−/−^ mice) [17]; this reduction might translate into impaired receptor-dependent signaling. We recently demonstrated that the stimulation of the A_2A_ receptor subtype (A_2A_R) can restore a normal phenotype in NPC1 cellular models as measured by the normalization of mitochondrial membrane potential (mMP) and cholesterol content [18,19,20]. Moreover, the treatment of NPC1^−/−^ mice with the compound T1-11 (able to increase the level of adenosine in the brain by inhibiting its transporter ENT1 and to weakly stimulate A_2A_R) significantly improved their cognitive deficits, reduced Purkinje neuron loss and sphingomyelin accumulation in the liver, and extended their survival [21]. Altogether, the above-mentioned data converge to strongly support an impairment in adenosine signaling; increasing its levels by inhibiting the transporter ENT1 may represent a new approach that could provide a benefit to both peripheral and CNS pathology. 

Among different ENT1 inhibitors clinically used for other pathologies, here we focused on dipyridamole (DIP). DIP is an old platelet aggregation inhibitor that was firstly introduced in 1959. Its mechanism of action is based on the blockade of ENT1 that induces elevated levels of extracellular adenosine and on the inhibition of the phosphodiesterase 5 (PDE5) enzyme, which increases intracellular levels of cyclic-3′,5′-adenosine monophosphate (cAMP) [22]. 

The aims of the present study were to characterize the adenosine homeostasis in in vitro and in vivo NPC1 models and to evaluate the effect of DIP in fibroblasts from NPC1 patients, which are considered a valuable tool for the identification of new therapeutic strategies [23]. Our results demonstrate that DIP effectively reduces intracellular cholesterol accumulation and rescues mMP depolarization; such effects are mediated by the activation of the adenosine A_2A_ receptors which, in turn, elicit the ERK1/2 kinase signal. 

## 2. Results

### 2.1. The Expression of Proteins Involved in the Regulation of Adenosine Levels Is Deregulated Both in Fibroblasts from Patients and in NPC1 Mice

**Figure 1:**
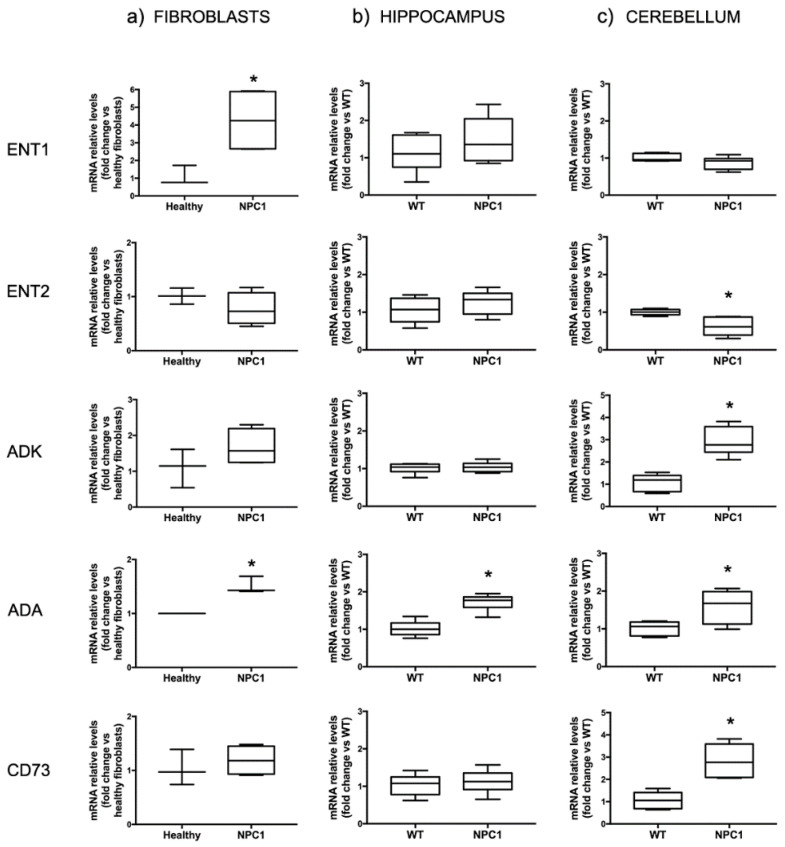
Expression of some major enzymes and transporters involved in the homeostatic control of extracellular adenosine levels. (**a**) mRNA levels of ENT1 and ADA are significantly increased in fibroblasts from NPC1 patients (*n* = 4 clones) with respect to levels found in healthy individuals (*n* = 3 clones). (**b**) mRNA levels of ADA are increased in the hippocampus of symptomatic NPC1 mice (PND60) with respect to their WT littermates (*n* = 6). (**c**) mRNA levels of ADK, ADA and CD73 are increased in the cerebellum of symptomatic NPC1 mice (PND60) with respect to their WT littermates (*n* = 6 animals), while ENT2 expression is decreased. Data are expressed as box-plots (whiskers from min to max values). * *p* < 0.05 after two-tailed Student’s *t*-test.

In order to characterize the adenosine metabolism in NPC1, we assessed by RT-PCR the gene expression of some major proteins that modulate extracellular adenosine amount. In particular, we analyzed the expression of the transporters ENT1 and 2, and of the enzymes adenosine kinase (ADK), adenosine deaminase (ADA) and ecto-5′-nucleotidase (CD73) in NPC1 human fibroblasts (Figure 1a) and in the hippocampus (Figure 1b) and cerebellum (Figure 1c) of symptomatic NPC1 mice (post-natal day 60, PND60). As can be seen in Figure 1a, we found a significant over-expression of ENT1 in NPC1 fibroblasts with respect to healthy fibroblasts (4.26 ± 0.92 vs. 1.1 ± 0.32, *p* < 0.05); ADA was also over-expressed in NPC1 cells (1.5 ± 0.09 vs. 1.0 ± 0.0, *p* < 0.05). On the contrary, ENT2 (0.77 ± 0.15 vs. 1.01 ± 0.08), ADK (1.67 ± 0.25 vs. 1.1 ± 0.3) and CD73 (0.99 ± 0.11 vs. 1.02 ± 0.21) were not differently expressed in NPC1 vs. healthy fibroblasts. In the hippocampus (Figure 1b), ENT1 (1.48 ± 0.25 vs. 1.11 ± 0.19), ENT2 (1.26 ± 0.12 vs. 1.05 ± 0.14), ADK (1.03 ± 0.05 vs. 1.00 ± 0.05) and CD73 (1.19 ± 0.14 vs. 1.03 ± 0.19) were not changed in NPC1 mice, while ADA expression was found significantly increased in NPC1 mice with respect to WT (1.72 ± 0.09 vs. 1.02 ± 0.08, *p* < 0.05). In the cerebellum (Figure 1c), ADK (2.92 ± 0.26 vs. 1.06 ± 0.17, *p* < 0.05), ADA (1.59 ± 0.18 vs. 1.01 ± 0.07, *p* < 0.05) and CD73 (2.8 ± 0.29 vs. 1.06 ± 0.16, *p* < 0.05) were increased, while ENT2 was decreased in NPC1 mice (0.63 ± 0.11 vs. 1.00 ± 0.03, *p* < 0.05); ENT1 was not changed (0.88 ± 0.07 vs. 1.00 ± 0.04). 

These results support the hypothesis that adenosine homeostasis is altered in NPC1 and that such an alteration could be due, at least in part, to the deregulation of enzymes and transporters that control extracellular and intracellular levels of adenosine.

### 2.2. Treatment with Dipyridamole Reduces Intracellular Cholesterol Accumulation in NPC1 Fibroblasts and Increases Mitochondrial Membrane Potential

**Figure 2:**
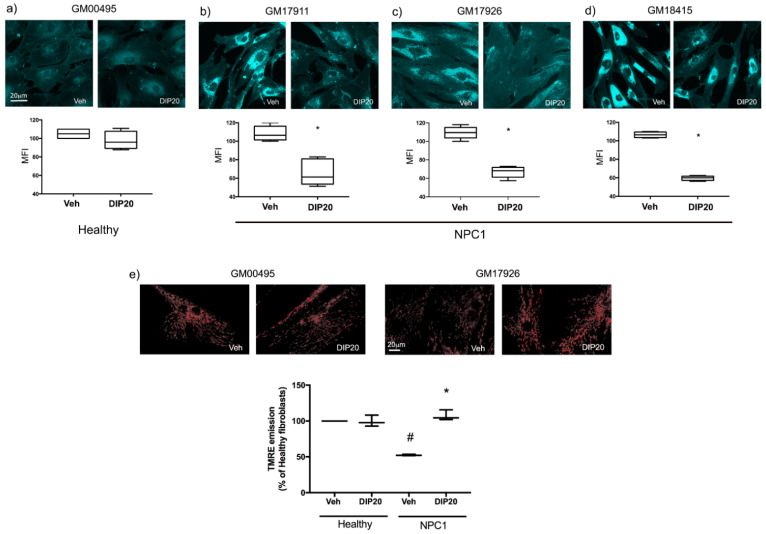
Effect of treatment with dipyridamole (DIP20: 20 μM over 48 h) on intracellular cholesterol distribution and mitochondrial membrane potential (mMP) in healthy and NPC1 fibroblasts. Filipin III was used to visualize unesterified cholesterol distribution in cells; TMRE fluorescence intensity was used to measure mMP. (**a**–**d**) Representative images of Filipin III-stained GM00495 healthy and GM17911, GM17926, and GM18415 NPC1 fibroblasts, respectively; the graphs below show the quantification of mean fluorescence intensity (MFI) of Filipin III staining, expressed as percentage of veh-treated cells. (**a**) DIP20 did not affect the uniform distribution of cholesterol in GM00495 healthy fibroblasts (*n* = 4 independent experiments). (**b**–**d**) DIP20 significantly reduced cholesterol accumulation in GM17911 (*n* = 6 independent experiments), GM17926 (*n* = 10 independent experiments) and GM18415 (*n* = 4 independent experiments) NPC1 fibroblasts, respectively. (**e**) Representative images of TMRE fluorescence analysis in GM00495 healthy and GM17926 NPC1 fibroblasts; the graph below shows the quantification of TMRE fluorescence intensity expressed as a percentage of veh-treated GM00495 cells. mMP is decreased in veh-treated GM17926 NPC1 fibroblasts but DIP20 restored its levels to GM00495 healthy cells (*n* = 3 independent experiments). * *p* < 0.05 vs. veh-treated NPC1 cells; # *p* < 0.05 vs. veh-treated healthy cells after two-tailed Student’s *t*-test in (**a**) and one-way ANOVA in (**b**). Data are expressed as box-plots (whiskers from min to max value). Scale bars = 20 µm.

In order to evaluate if enhancing extracellular levels of adenosine could ameliorate the pathological phenotype of NPC1 fibroblasts, we treated cells with 20 μM of dipyridamole (DIP20); after 48 h we analyzed the effect elicited on the accumulation of unesterified cholesterol by measuring the mean fluorescence intensity (MFI) of Filipin III staining. As shown in Figure 2, DIP20 reduced cholesterol accumulation in all the NPC1 clones tested. In particular, the following results, expressed as percentages of veh-treated cells in the underlying graphs, were obtained in NPC1 fibroblasts: GM17911 (Figure 2b), 65.3 ± 5.4 in DIP20-treated cells vs. 108.3 ± 3.2 in veh-treated cells (*p* < 0.01); GM17926 (Figure 2c), 66.7 ± 1.8 in DIP20-treated cells vs. 109.3 ± 1.9 in veh-treated cells (*p* < 0.0001); GM18415 (Figure 2d), 59.7 ± 1.3 in DIP20-treated cells vs. 106.5 ± 1.5 in veh-treated cells (*p* < 0.05). On the contrary, DIP20 had no effects in the healthy fibroblasts GM00495 (Figure 2a; 97.6 ± 4.9 in DIP20-treated cells vs. 105.0 ± 2.9 in veh-treated cells).

Given that depolarization of the mitochondrial membrane potential (mMP) has been described in NPC1 fibroblasts by our group [18], we decided to evaluate if DIP20 treatment could ameliorate this pathological hallmark of the disease. To this aim we used the video-imaging technique to measure the fluorescence emitted by the potential-sensitive and lipophilic dye TMRE in GM17926 NPC1 and GM00495 healthy fibroblasts (Figure 2e). As can be seen in the underlying graph (data are expressed as percentages of TMRE fluorescence intensity in healthy cells), the deficit found in NPC1 fibroblasts (52.4 ± 0.56 in veh-treated NPC1 vs. 99.94 ± 0.0083 in veh-treated GM00495 healthy fibroblasts) was completely reverted by the treatment with dipyridamole (52.4 ± 0.56 in veh-treated vs. 107.32 ± 4.21 in DIP20-treated GM17926 clone). Dipyridamole did not influence this parameter in the healthy fibroblast (99.94 ± 0.0083 in veh-treated vs. 99.58 ± 4.48 in DIP20-treated cells).

These results support the hypothesis that the inhibition of ENT1 in NPC1 can revert different pathological deficits and, thus, it could represent a valuable therapeutic strategy for the disease.

### 2.3. Dipyridamole Rescues NPC1-Related Phenotypes in Fibroblasts by an A_2A_R-Dependent Pathway

In order to investigate which mechanism is involved in the effect of dipyridamole on cholesterol accumulation and mMP, we wanted to verify if its application increased adenosine levels in NPC1 fibroblasts. As expected, and in line with [24], after 3 h of treatment with DIP, a significant increase in adenosine levels could be found in the supernatants (Figure S1, 105.3 ± 2.4 in veh-treated cells vs. 138.1 ± 10.7 in DIP20-treated cells).

**Figure 3:**
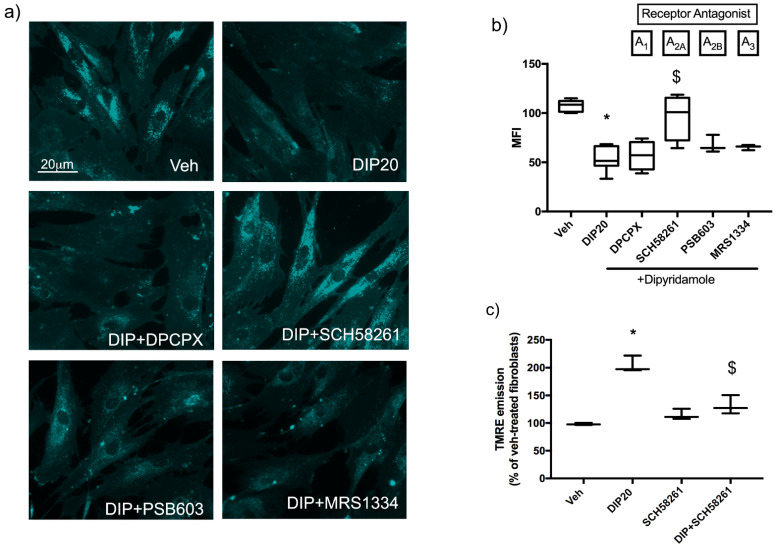
Effect of adenosine receptors inhibition on dipyridamole-mediated reduction in cholesterol accumulation and rescue of mMP. (**a**) Representative images of Filipin III-stained NPC1 fibroblasts (GM17926) treated with 20 μM of dipyridamole (DIP20) alone or in combination with A_1_R (15 μM DPCPX), A_2A_R (10 μM SCH58261), A_2B_R (500 nM PSB603) and A_3_R (1 μM MRS1334) antagonists. Scale bar = 20 µm. (**b**) Quantification of mean fluorescence intensity (MFI) of Filipin III staining, expressed as percentage of veh-treated cells; the A_2A_R antagonist SCH58261 inhibited the effect of DIP20 on cholesterol accumulation (*n* = 3–8 independent experiments). (**c**) Quantification of mean fluorescence intensity (MFI) of TMRE emission, expressed as percentage of veh-treated cells. The A_2A_R antagonist SCH58261 inhibited the effect of DIP20 on mMP (*n* = 3 independent experiments). * *p* < 0.05 vs. veh-treated; $ *p* < 0.05 vs. DIP20-treated cells after one-way ANOVA. Data are expressed as box-plots (whiskers from min to max values).

RT-PCR screening of NPC1 fibroblasts indicated that they express all adenosine receptors (data not shown); thus, to assess if dipyridamole effect could be mediated by adenosine-dependent activation of its receptors, we co-treated cells with dipyridamole and the following selective antagonists: DPCPX, SCH58261, PSB603 and MRS1334 to block A_1_, A_2A_, A_2B_ and A_3_ receptors, respectively. The effect of all antagonists was evaluated on cholesterol accumulation (Figure 3a,b); as can be seen in the graph (Figure 3b) only the A_2A_R antagonist SCH58261 was able to completely counteract the effect of dipyridamole (96.27 ± 11.59 in DIP + SCH58261 vs. 53.82 ± 4.22 in DIP20, *p* < 0.05); DPCPX (56.89 ± 7.29 in DIP + DPCPX vs. 53.82 ± 4.22 in DIP20), PSB603 (67.83 ± 5.18 in DIP + PSB603 vs. 53.82 ± 4.22 in DIP20), and MRS1334 (65.27 ± 1.54 in DIP + MRS1334 vs. 53.82 ± 4.22 in DIP20) did not affect the reduction in cholesterol accumulation induced by dipyridamole treatment (53.82 ± 4.22 in DIP20 vs. 107.5 ± 1.95 in veh-treated cells, *p* < 0.05). SCH58261 also blocked the increase in mMP induced by dipyridamole (Figure 3c, 131.8 ± 9.8 in DIP+SCH58216 vs. 204.8 ± 8.48 in DIP20-treated cells).

These results demonstrate that the adenosine increase induced by dipyridamole stimulates the A_2A_R subtype which, in turn, reduces the accumulation of intracellular cholesterol and rescues the depolarization of mMP. 

### 2.4. Functional Impact of cAMP/PKA and MAPK Pathways on Dipyridamole-Dependent Reduction of Cholesterol Accumulation

**Figure 4:**
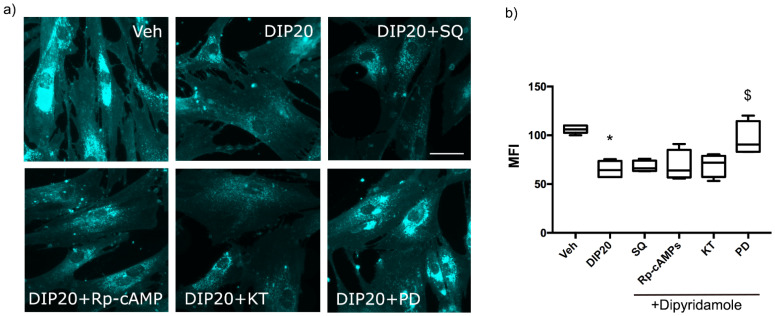

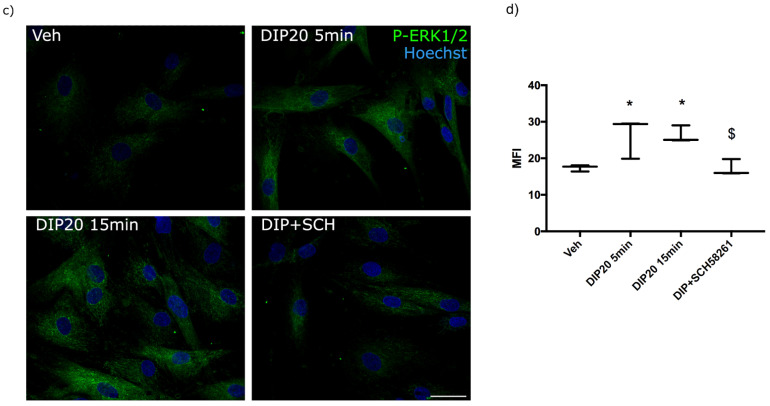
Effect of inhibitors of A_2A_R-dependent pathways on the ability of dipyridamole to reduce cholesterol accumulation. (**a**) Representative images of Filipin III-stained NPC1 fibroblasts (GM17911) treated with 20 μM of dipyridamole (DIP20) alone or in combination with an adenylate cyclase inhibitor (100 μM SQ22536), an inhibitor of cAMP-dependent activation of PKA (200 μM Rp-cAMPs), an inhibitor of PKA (10 μM KT5720) and an inhibitor of MEK1 (50 μM PD98059). (**b**) Quantification of mean fluorescence intensity (MFI) of Filipin III staining, expressed as a percentage of veh-treated cells. The MEK1 inhibitor PD98059 blocked the effect of DIP20 on cholesterol accumulation. (**c**) Representative images of P-ERK1/2 and Hoechst-stained NPC1 fibroblasts (GM17911) treated for 5 and 15 min with 20 μM of dipyridamole (DIP20 5 min; DIP20 15 min) alone or in combination with the A_2A_R antagonist SCH58261 (10 μM). (**d**) Quantification of mean fluorescence intensity (MFI) of P-ERK1/2 staining. * *p* < 0.05 vs. veh-treated; $ *p* < 0.05 vs. DIP20-treated cells after one-way ANOVA (*n* = 3–5 independent experiments). Data are expressed as box-plots (whiskers from min to max values). Scale bars = 20 µm.

In an attempt to better elucidate the signaling pathway elicited by A_2A_R to rescue the NPC1 phenotype, we used pharmacological tools to inhibit different intracellular signal transduction systems and evaluated their impact on the dipyridamole-dependent reduction of cholesterol accumulation. Given that the stimulation of A_2A_R can activate adenylate cyclase (AC) to increase intracellular amount of cyclic AMP (cAMP) which, in turn, activates PKA, we inhibited the activity of AC by treating cells with the selective inhibitor SQ22536; as can be seen in Figure 4, SQ22536 (SQ) did not revert the reduction in intracellular cholesterol accumulation induced by dipyridamole (Figure 4b, 67.9 ± 2.93 in DIP20 + SQ treated cells vs. 65.2 ± 3.73 in DIP20-treated cells). These results indicate that dipyridamole activity is not dependent on new-generated cAMP by A_2A_R-mediated activation of AC. However, it should be recalled that dipyridamole is also a phosphodiesterase inhibitor and, as such, it can increase intracellular cAMP by reducing its degradation; thus, it cannot be excluded that the effect of A_2A_R on NPC1 phenotype could rely on the activation of PKA mediated by the increase of cAMP due to the inhibitor action of dipyridamole onto phosphodiesterase. In order to further exclude the involvement of cAMP signaling in dipyridamole’s effect, we blocked the cAMP-dependent activation of PKA by the cell-permeable inhibitor Rp-cAMPs; Rp-cAMPs was not able to revert the effect of dipyridamole on cholesterol accumulation (Figure 4b, 68.59 ± 7.86 in DIP20 + Rp-cAMP treated cells vs. 65.2 ± 3.73 in DIP20-treated cells) even when used at 500 μM or in combination with SQ22536 (data not shown). These results were confirmed by the application of another PKA inhibitor, KT5720 (Figure 4b, 69.38 ± 8.75 in DIP20+KT treated cells vs. 65.2 ± 3.73 in DIP20 treated cells). On the contrary, the MEK inhibitor PD98059 completely reverted the reduction of cholesterol induced by dipyridamole (Figure 4b, 95.9 ± 8.75 in DIP + PD treated cells vs. 65.2 ± 3.73 in DIP20 treated cells, *p* < 0.05). In order to confirm the activation of ERK1/2 signaling by A_2A_R engagement, we treated NPC1 fibroblasts with 20 µM dipyridamole for 5 and 15 min and evaluated the expression of the phosphorylated form of ERK1/2 (P-ERK1/2) by immunofluorescence (Figure 4c,d). As can be seen in Figure 4d, dipyridamole significantly increased the phosphorylation of ERK1/2 both after 5 min (26.25 ± 3.2 in DIP20 5 min vs. 17.4 ± 0.5 in Veh) and 15 min (26.32 ± 1.3 in DIP20 15 min vs. 17.4 ± 0.5 in Veh); the effect of dipyridamole was blocked by the A_2A_R antagonist SCH58261 (17.2 ± 1.3 in DIP+SCH vs. 26.32 ± 1.3 in DIP20 15 min and 26.25 ± 3.2 in DIP20 5 min).

These results indicate that cholesterol reduction mediated by dipyridamole activation of A_2A_R is elicited by the activation of ERK1/2 kinases. 

## 3. Discussion

In the present paper, we hypothesized that a dysfunction of adenosine homeostasis can occur in NPC and that the inhibition of its transporter ENT1 could be beneficial. The first evidence that adenosine homeostasis could be disrupted in NPC comes from the observation by Zhou and co-workers of a decreased tonic inhibition of synaptic transmission in hippocampal slices from NPC1 mice due to a reduction in the levels of basal extracellular adenosine [17]. The authors hypothesized that the mutation in NPC1 protein could be responsible not only for the impaired trafficking of cholesterol to the plasma membrane but also for a deficit in exocytosis of ATP (a major source of extracellular adenosine in the brain) that can be responsible for the observed decrease in adenosine. However, other mechanisms can be involved in the reduction of its levels; in fact, the expression of enzymes or transporters involved in adenosine metabolism could be abnormally regulated in NPC, as already demonstrated in many neurodegenerative diseases [25]. In order to test this hypothesis, we analyzed the mRNA expression of proteins involved in the control of adenosine homeostasis in both fibroblasts from patients and brain regions from symptomatic NPC1 mice; in particular, we analyzed the hippocampus and the cerebellum because they are severely affected in NPC, being characterized by atrophy and neuronal degeneration, respectively [5]. Our results revealed that the expression of ADA is increased both in NPC1 fibroblasts and in the above brain regions from mice; particularly interesting are the data obtained in fibroblasts because they are in line with the increased expression of ADA observed in a quantitative proteomic study performed in NPC1 fibroblasts [26]. Concerning the remaining proteins, we found increased expression of mRNAs for ADK and CD73 only in the cerebellum, ENT1 increased in fibroblasts and ENT2 slightly, but significantly, reduced in the cerebellum. Overall, these data are indicative of an abnormal adenosine homeostasis in NPC1, which could contribute to the reduced adenosine levels observed in the hippocampus of mice [17]. Very interestingly, the deregulated expression of proteins involved in the regulation of adenosine levels found also in fibroblasts from patients, makes us suppose that an abnormal adenosine metabolism could also occur in patients and that a deeper comprehension of its role in the disease would be a worthwhile endeavor (for example by measuring adenosine levels in patient plasma and expression of adenosine-regulator proteins in the brain). After all, accumulating evidence implicates dysregulated adenosine metabolism in many neurodegenerative diseases; for example, an up-regulation of ADA, ADK and CD73 has been found both in patients and in mouse models of Alzheimer’s disease (AD) [25,27,28,29]; ENT1, ENT2 and ADA were found increased in Huntington’s disease (HD) [16]. Interestingly, the pharmacological suppression of ENT1 improved symptoms in animal models of either AD [15,25] and HD [16], indicating that the manipulation of this transporter could represent a therapeutic approach to treat neurodegenerative disorders characterized by a deregulation of adenosine homeostasis, like that occurring in NPC. Indeed, in our previous paper we demonstrated that long-term treatment with the adenosine analog T1-11 that not only activates A_2A_R but also elevates extracellular adenosine levels in the brain by inhibiting ENT1, showed beneficial effects in a mouse model of NPC1 [21]. T1-11 is not a clinically-approved drug but several compounds with ENT1 inhibitory activity are used in the clinic to treat different pathologies (e.g., ticagrelor, dilazep, sulindac, dipyridamole) [30,31,32]. Considering that repositioning a well-known drug represents an enormous advantage for patients both in terms of time (traditional programs for drug development typically take 10–17 years, while a repurposing approach allows a fast translation to the clinical setting) and of safety (they have known safety profile, pharmacokinetic and pharmacodynamic properties), here we focused on dipyridamole (DIP) because, compared to other ENT1 inhibitors, it presents the advantage to increase adenosine levels also in the brain [32]; in fact, it has been already tested in CNS pathologies such as schizophrenia [33] and restless legs syndrome [32].

Dipyridamole is an antithrombotic medication known to potentiate adenosine receptor-mediated signaling by either increasing extracellular concentration of adenosine (through the inhibition of ENT1/2 transporter) and by interfering with the breakdown of cAMP to AMP (through the inhibition of phosphodiesterase) [34]. In order to obtain a proof of concept that increasing adenosine receptor signaling could be beneficial in NPC, we treated fibroblasts derived from NPC1patients with dipyridamole; the efficacy of the treatment was evaluated on its ability to impact intracellular cholesterol accumulation and on mitochondrial membrane potential (mMP) decrease, both representing hallmarks of pathology. Our results clearly demonstrate that dipyridamole is able to decrease cholesterol accumulation in all four NPC1 clones tested while not influencing the distribution of cholesterol in fibroblasts from a healthy donor. Moreover, dipyridamole also rescued the depolarization of mMP in NPC1 fibroblasts without affecting the mMP in healthy ones. These data support our hypothesis that repurposing dipyridamole in NPC could represent a valid therapeutic approach for this devastating disease. In an attempt to shed light on the cellular mechanism elicited by dipyridamole to reduce the pathological hallmarks of the disease, we used pharmacological tools to inhibit the cellular pathways most likely responsible for its effects. In fact, as stated above, dipyridamole can increase either extracellular adenosine levels by the inhibition of ENT1/2-mediated uptake and intracellular cAMP by phosphodiesterase inhibition [34]. Indeed, we verified that dipyridamole treatment induced an increase of adenosine amount in the supernatant of NPC1 fibroblasts as already demonstrated for other cellular systems [24]. Thus, we investigated the possibility that extracellular adenosine could mediate the effect of dipyridamole through the activation of its receptors A_1_, A_2A_, A_2B_ and A_3_. We blocked each subtype with selective antagonists in presence of dipyridamole in order to evaluate if a reversion of dipyridamole effect could occur. The only antagonist that counteracted the effect of dipyridamole on cholesterol accumulation was the A_2A_ receptor antagonist SCH58261. Moreover, the blockade of A_2A_ receptor was also able to revert dipyridamole-mediated increase in mMP. We are confident that the concentration of each antagonist used in our experiments was sufficient to block each receptor; in fact, 15 µM DPCPX and 10 µM SCH58261 were already employed to block A_1_R and A_2A_R in astrocytes [35] while A_2B_R (500 nM PSB603) and A_3_R (1 μM MRS1334) antagonists were used at the highest concentration that was found non-toxic for cells. These data demonstrate that dipyridamole treatment of NPC1 fibroblasts increases extracellular levels of adenosine, which, in turn, stimulates the A_2A_R subtype to reduce cholesterol accumulation and increase mMP.

The most commonly recognized signal transduction mechanism for A_2A_Rs is the activation of adenylate cyclase (AC) by means of G_s_ protein [14,36,37,38]; the activation of AC generates an increase in intracellular cAMP and the subsequent activation of PKA [37]. In addition to this well-characterized conventional signaling pathway, A_2A_R can also trigger MAPK activation by a G protein- and cAMP-independent action [37]. Moreover, A_2A_R signaling is deeply affected by lipid environment of plasma membrane since a bulk membrane cholesterol depletion, like that occurring in NPC [39,40,41,42,43], inhibits the A_2A_R signaling mediated by cAMP without affecting its ability to activate MAPKs [44,45]. Our group already demonstrated that the stimulation of A_2A_R in NPC1 fibroblasts by its agonist CGS21680 induced an activation of the MAPK ERK1/2 in a PKA-independent manner [18]. In order to investigate the signaling cascade triggered by the A_2A_R activation induced by the dipyridamole-dependent increase in extracellular adenosine, NPC1 fibroblasts were incubated with inhibitors of AC and MAPKs. The results obtained demonstrate that the inhibition of AC by SQ22536 is not enough to revert the effect of dipyridamole on cholesterol; however, considering that dipyridamole can increase intracellular cAMP by inhibiting phosphodiesterase activity [34], we wanted to exclude that the lack of effect of SQ22536 could be due to an elevation of cAMP caused by its reduced breakdown that could be sufficient to activate PKA. For this reason, we blocked the activation of PKA with a pan-PKA inhibitor (KT5720) and with a cAMP-dependent inhibitor (Rp-cAMPs); in both conditions, the effect of dipyridamole on cholesterol accumulation was not counteracted. These results lead us to conclude that the effect of dipyridamole is not mediated by cAMP; on the contrary, the inhibition of the ERK1/2 pathway by PD98059 completely reverted the effect of dipyridamole on cholesterol accumulation. Moreover, immunofluorescence analysis of ERK1/2 activation confirmed that dipyridamole induced the phosphorylation of ERK1/2, which was abolished by the A_2A_R antagonist SCH58261. Evidence in the literature already demonstrated that dipyridamole can exert its activity in a cAMP-independent way [46]; this interesting paper investigated the mechanism of action of dipyridamole for its known ability to inhibit lipogenic gene expression by blocking the maturation of sterol regulatory element-binding proteins (SREBPs); SREBPs are key regulators of the expression of genes coding for enzymes required for the synthesis of triglycerides and cholesterol. In their paper, the authors concluded that the above-mentioned effect was not depending on cAMP, but they did not investigate other cellular signaling (e.g., that elicited by A_2A_R). On the basis of these results, we can speculate that the activation of A_2A_Rs due to dipyridamole-mediated increase in extracellular adenosine could be responsible for the inhibition of the SREBP pathway, which, indeed, is found overactivated in fibroblasts from NPC patients [42,43]; thus, the beneficial effects of dipyridamole on cholesterol accumulation could rely on its ability to inhibit SREBPs maturation that can be responsible for a reduced de novo synthesis of cholesterol. Further studies investigating this hypothesis are worth considering. Taken together, our results demonstrate that dipyridamole treatment of NPC1 fibroblasts ameliorates their pathological phenotype by the activation of A_2A_Rs induced by the extracellular release of adenosine; A_2A_R engages the MAPK signaling cascade to exert its effect. 

In conclusion, our study confirms that targeting the adenosine signaling could represent a new therapeutic strategy for NPC disease; the observation that increasing adenosine signaling with dipyridamole phenocopies the effect observed with the use of the selective A_2A_R agonist CGS21680 in NPC1 fibroblasts [18] paves the way for further characterization of this drug in in vivo models of the disease.

## 4. Materials and Methods

### 4.1. Drugs

Dipyridamole (cat#D9766, Sigma-Aldrich, Merk Life Science, Milan, Italy); 2-(2-Furanyl)-7-(2-phenylethyl)-7*H*-pyrazolo[4,3-e][1,2,4]triazolo[1,5-*c*]pyrimidin-5-amine, SCH58261 (cat#2270, Tocris, Bio-Techne, Milan, Italy); 8-Cyclopentyl-1,3-dipropylxanthine, DPCPX (cat#0439, Tocris, Bio-Techne, Milan, Italy); 8-[4-[4-(4-Chlorophenzyl)piperazide-1-sulfonyl)phenyl]]-1-propylxanthine, PSB603 (cat#3198, Tocris, Bio-Techne, Milan, Italy); 1,4-Dihydro-2-methyl-6-phenyl-4-(phenylethynyl)-3,5-pyridinedicarboxylic acid 3-ethyl-5-[(3-nitrophenyl)methyl] ester, MRS1334 (cat#1385, Tocris, Bio-Techne, Milan, Italy); KT5720 (cat#K3761, Sigma, Merk Life Science, Milan, Italy); PD98059 (car#513001, Calbiochem, Merk Life Science, Milan, Italy); 9-(Tetrahydro-2-furanyl)-9*H*-purin-6-amine, SQ22536 (cat#1453, Tocris, Bio-Techne, Milan, Italy); (*R*)-Adenosine, cyclic 3′,5′-(hydrogenphosphorothioate) triethylammonium, Rp-cAMPs (cat#1337, Tocris, Bio-Techne, Milan, Italy).

### 4.2. Cell Cultures

The following human fibroblasts from individuals of either sex were obtained from the NIGMS Human Genetic Cell Repository at the Coriell Institute for Medical Research (Camden, NJ, USA): healthy fibroblast clones: GM00043, GM00495; NPC1-mutant fibroblast clones: GM17911 characterized by the missense mutations ile1061thr in allele 1 and thr1036met in allele 2, GM17926 characterized by the missense mutations ile1061thr in allele 1 and tyr509ser in allele 2, GM17924 with two identified mutations (the deletion mutation 451_452delAG in allele 1 and the missense mutation tyr825cys in allele 2) and GM18415 with a cys74tyr missense mutation in allele 1 (the mutation in allele 2 has not been identified); the healthy fibroblast NHDF-A was obtained from Lonza (Lonza Group, Basel, Switzerland). Cells were grown at 37 °C in 5% CO_2_ in Eagle’s Minimum Essential Medium containing 2 µM L-glutamine, 15% fetal bovine serum, 1% of non-essential amino-acids and 1 U/mL penicillin/streptomycin. Each clone was exposed to dipyridamole and analyzed for cholesterol accumulation after Filipin III staining (see Section 4.5 for details); given the comparable responses obtained, subsequent functional experiments were performed with GM00495, GM17911 and GM17926 because of their better growing ability with respect to the remaining clones.

### 4.3. Mice

Breeding pairs of BALBc heterozygous (NPC^+/−^) mice were purchased from Charles-River Laboratories (Calco, Italy) and bred to generate homozygous (NPC1^−/−^, hereafter indicated as NPC1 mice) and control (WT) mice. Genotyping was performed as previously described [21]. Mice were kept under standardized temperature, humidity, and lighting conditions and had free access to water and food. Animal facilities were under regular supervision by resident veterinarians. NPC1 and WT littermates were sacrificed in a symptomatic stage of the disease (post-natal day 60, PND60) and brain regions were collected and store at −80 °C until RT-RNA experiments. All animal procedures were carried out according to the European and Italian legislation (Directive 2010/63/EU, L.D. No. 26/2014), and approved by the Italian Ministry of Health (894/2020-PR of September 2020).

### 4.4. Real-Time Quantitative Polymerase Chain Reaction (RT-PCR)

Dissected hippocampi, cerebellums and fibroblast pellets were homogenized in TRIzol Reagent (Sigma, Merk Life Science, Milan, Italy) and RNA was extracted using the Trizol/chloroform method followed by an isopropanol precipitation. The RNA pellet was washed once in 75% ethanol, let dry, then eluted in Nuclease-free water. Samples were dosed using the NanoDrop 2000 (ThermoFisher Scientific, Milan, Italy). Total RNA (1 µg) from each sample was transcribed into complementary DNA using the High-Capacity cDNA Reverse Transcription Kit (Applied Biosystems, ThermoFisher Scientific, Milan, Italy), according to the manufacturer’s instructions. Real-time PCR was performed on the reverse transcription products with SYBR™ Green master mix (Applied Biosystems, ThermoFisher Scientific, Milan, Italy) using an ABI Prism 7500 Sequence Detection System (Applied Biosystems, ThermoFisher Scientific, Milan, Italy). Annealing temperature was 60 °C for all the primer pairs listed.

All samples were run in duplicate, and each PCR well contained 20 μL as a final volume of reaction, including 2 μL complementary DNA corresponding to approximately 60 ng total RNA, 750 nM of each primer, and 10 μL PCR master mix. Thermal cycling conditions were as follows: 1 cycle at 95 °C for 10 min, 40 cycles at 95 °C for 15 s, and 60 °C for 1 min. The relative expression level of each mRNA was calculated using the 2^−ΔΔCt^ method, normalized to β-actin, and relative to the control group (i.e., hippocampus or cerebellum of WT mice or healthy fibroblasts). The amplification specificity was verified by melting curve analyses. Primers for human and mouse ENT1, ENT2, ADK, ADA, CD73, β-actin and for human A_1_, A_2A_, A_2B_ and A_3_ were from Integrated DNA Technologies (IDT, TEMA Ricerca, BO, Italy). Primers used in this study are listed in Table S1 (Supplementary Materials).

### 4.5. Cell Treatments

Cells were plated on coverslips at a density of 20,000 cells/500 μL in 24-well plates and after 24 h they were treated with the different drugs. Treatments with dipyridamole (20 μM) were performed for 48 h; this concentration was chosen on the basis of plasma exposure achieved in humans after oral consumption as described in [47]. SCH58261 (A_2A_ antagonist, 10 μM), DPCPX (A_1_ antagonist, 15 μM), PSB603 (A_2B_ antagonist, 500 nM), MRS1334 (A_3_ antagonist, 1 μM), KT5720 (PKA inhibitor, 10 μM), PD98059 (MEK 1 inhibitor, 50 μM), SQ22536 (adenylate cyclase inhibitor, 100 μM), Rp-cAMPs (inhibitor of cAMP-induced activation of PKA, 200 μM) were applied 30 min before and then along with dipyridamole. KT5720 was dissolved in methanol (MetOH) and Rp-cAMPs in distilled H_2_O. All the remaining drugs were dissolved in dimethyl-sulfoxide (DMSO). In vehicle-treated cells, an amount of DMSO, H_2_O or MetOH equal to the volume present in treated-cells was added. After 3 h cells were used for adenosine quantification and after 48 h they were used for Filipin III and TMRE analyses. For the evaluation of P-ERK1/2 expression, cells were treated for 5 and 15 min with dipyridamole (20 μM) as in [18].

### 4.6. Measurement of Adenosine Levels in Culture Supernatants

Adenosine levels were quantified according to the method described in [24]. In particular, after 3 h of incubation with dipyridamole, supernatants were collected, centrifuged at 9000× *g* for 5 min to remove cell debris and assayed by using the Adenosine Assay kit MET-5090 (Cell Biolabs, Inc., San Diego, CA, USA).

### 4.7. Filipin III Fluorescence and P-ERK1/2 Immunofluorescence

Cells were grown onto coverslips and were fixed in 4% formaldehyde in PBS for 15 min at room temperature (RT). For Filipin III staining, after washing in PBS, cells were incubated with 250 mg/mL Filipin III for 1 h at RT in the dark. For P-ERK1/2 analysis, after washing with PBS, they were permeabilized in 0.2% Triton X-100 in PBS for 5 min at RT and incubated with anti-P-ERK1/2 (1:100 in PBS; cat#4370, Cell Signaling Technology, Milan, Italy) antibody overnight at +4 °C. Cells were washed with PBS and incubated for 45 min at 37 °C with a goat anti-rabbit Alexa Fluor 488-conjugated antibody (1:200 in PBS; cat#A11008; Invitrogen, ThermoFisher Scientific, Milan, Italy); nuclei were counterstained with Hoechst 33258 (Sigma-Aldrich, Merk Life Science, Milan, Italy).

Coverslips were mounted with PBS-Glycerol (1:1) and observed using an Eclipse 80i Nikon (Amsterdam, The Netherlands) Fluorescence microscope equipped with a VideoConfocal (ViCo) system. Filipin fluorescence was observed with UV excitation around 360 nm and emission around 480 nm. Fluorescence quantification was conducted using NIH ImageJ software (https://imagej.nih.gov/ij/download.html; accessed on 20 March 2022) and by means of threshold fluorescence intensity analysis within a region of interest, corresponding to a single cell profile. At least ten different fields in each coverslip were captured for a total of 40–50 cells analyzed in each independent experiment. Filipin III staining is considered a semi-quantitative technique and its mean fluorescence intensity (MFI) was expressed as the percentage of each treatment group over the control one (i.e., veh-treated cells); for P-ER1/2 they are expressed as mean MFI. Data were collected from a minimum of 3 independent experiments for each condition and shown in bar graphs as mean ± SEM.

### 4.8. Mitochondrial Inner Membrane Potential Measurement

To measure mitochondrial membrane potential (mMP), the fluorescence video-imaging technique with the potentiometric dye tetramethylrhodamine ethyl ester perchlorate (TMRE) (Cat#87917 Lot#1350313 Sigma-Aldrich, Merk Life Science, Milan, Italy) was used. Cell loading was achieved by incubation with TMRE (30 nM for 30 min, at RT in dark condition). The presence of the dye was maintained throughout the experiment to prevent dye leakage. Loading and experimental solution bathing the cells in static condition had the following composition (mM): 140 NaCl, 5 KCl, 2.5 CaCl_2_, 1 MgCl_2_, 10 glucose, 10 Hepes, pH 7.4. A 535 nm excitation wavelength was produced by means of a monochromator (Polychrome II; Till Photonics, Munich, Germany) and directed to the culture through an optic fiber. The TMRE emission above 590 nm was collected through an oil immersion objective (Olympus: 40×, 1.35 NA) and recorded by a CCD cooled digital camera (PCO; Sensicam, Kelheim, Germany) mounted on an inverted microscope (Axiovert 135, Zeiss; Oberkochen, Germany). Recording and first analysis of the data were made possible using Imaging Workbench 6.0 software package (Indec BioSystems, Santa Clara, CA, USA). For further analysis and graphical presentation, the Origin 7.5 software package was used (OriginLab, Northampton, MA, USA). mMP of individual mitochondria were calculated as the average intensity of at least two points along line profiles crossing mitochondria. The TMRE fluorescence intensity was normalized as the percentage of each treatment group over the control. Data were collected from a minimum of 3 independent experiments for each condition and shown in bar graphs as mean ± SEM.

### 4.9. Statistical Analysis

Data are expressed as means ±SEM. Statistical significance was evaluated using two-tailed unpaired Student’s *t* test was used to compare difference between two groups. One-way ANOVA followed by Tukey’s multiple comparisons test was used for comparison of multiple groups. Differences were considered statistically significant when *p* < 0.05. Analyses were performed using StataTm 8.1 software (Stata, College Station, TX, USA) and GraphPad Prism Software (San Diego, CA, USA).

## Data Availability

All data generated and analyzed are included in the published article, Supplementary Materials and upon reasonable request are available from the corresponding author.

## Supplementary Materials

The following are available online at https://www.mdpi.com/article/10.3390/ijms23073456/s1.
