# Supplementary material for: Repurposing Dipyridamole in Niemann Pick Type C Disease: A Proof of Concept Study
Source: Int J Mol Sci. 2022 Mar 22;23(7):3456. doi: 10.3390/ijms23073456 (PMC8999038; doi:10.3390/ijms23073456)
Supplement: Supplementary file 1 [file ijms-23-03456-s001.zip › ijms-1643037-supplementary.pdf]

NAME	SEQUENCE (5′ to 3′)
hCD73 F<br>hCD73 R	GAAGGCCTTTGAGCATAGCG<br>CGACACTTGGTGCAAAGAACA
hADK F<br>hADK R	TGCCCTAATTGCTTCCTGAG<br>TTGGCATTTAAGTGGCACTATC
hENT1 F<br>hENT1 R	GGCCCAAGAAAGTGAAGCCA<br>ACCACTCAGGATCACCCCTG
hENT2 F<br>hENT2 R	CTTCCATACCCACTCTCTCACC<br>GAGAGAGAGGGGATTGGGTC
hADA F<br>hADA R	GGCTAACTACTCGCTCAACA<br>CGCATTGATGTTCAGCCTTT
hbeta-actin F<br>hbeta-actin R	CGAGGCCCAGAGCAAGAGAG<br>CTCGTAGATGGGCACAGTGTG
mENT1 F<br>mENT1 R	ATC AAT CAT GCG AAA GCA<br>GCA GGT GAA GAC CAG CAA
mENT2 F<br>mENT2 R	CAT GGA AAC TGA GGG GAA GA<br>GTT CCA AAG GCC TCA CAG AG
mADA F<br>mADA R	GAA GGC AAA GGA GGG CGT GGT CTA<br>GAT GTC CAC AGC CTC ACG CAC AAC
mADK F<br>mADK R	GTG CTA TTT GGA ATG GGG AAT<br>CAA CCA CTG AGC CAC TTT CAT
mCD73 F<br>mCD73 R	CAA ATC CCA CAC AAC CAC T<br>TGC TCA CTT GGT CAC AGG AC
mbeta-actin F<br>mbeta-actin R	AGGCATCCTCACCCTGAAGTA<br>CACACGCAGCTCATTGTAGA
hA1R F<br>hA1R R	TCCATCTCAGCTTTCCAGGC<br>CTCGAACTCGCACTTGATCAC
hA2AR F<br>hA2AR R	ACCTGCAGAACGTCACCAAC<br>TCTGCTTCAGCTGTCGTCGC
hA2BR F<br>hA2BR R	CACAGGACGCGCTGTACGTG<br>TTCTGTGCAGTTGTTGGTGG
hA3R F<br>hA3R R	AACGTGCTGGTCATCTGCGTGGTC<br>GTAGTCCATTCTCATGACGGAAAC

Table S1. Sequence of primers used in RT-PCR experiments. F: forward; R: reverse; h: human; m: mouse.

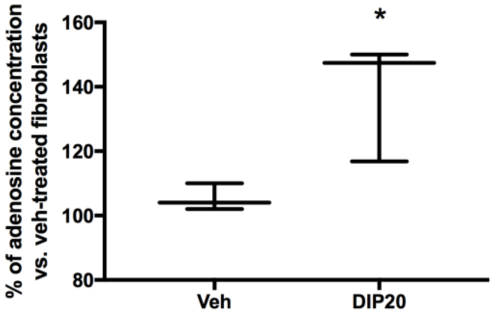

Figure S1. Effect of treatment with 20 μM of dipyridamole for 3h (DIP20) on extracellular adenosine levels in NPC1 GM17926 fibroblasts (n=3). As expected, DIP20 significantly increased the levels of extracellular adenosine measured in supernatants. *p<0.05 after Student’s t-test. Data are expressed as the percentage of adenosine measured in veh-treated cells; they are represented as box-plots (whiskers from min to max value).
